# Increased Probability of Co-Occurrence of Two Rare Diseases in Consanguineous Families and Resolution of a Complex Phenotype by Next Generation Sequencing

**DOI:** 10.1371/journal.pone.0146040

**Published:** 2016-01-20

**Authors:** Dennis Lal, Bernd A. Neubauer, Mohammad R. Toliat, Janine Altmüller, Holger Thiele, Peter Nürnberg, Clemens Kamrath, Anne Schänzer, Thomas Sander, Andreas Hahn, Michael Nothnagel

**Affiliations:** 1 Cologne Center for Genomics, University of Cologne, 50931, Cologne, Germany; 2 Department of Neuropediatrics, University Medical Faculty Giessen and Marburg, 35392, Giessen, Germany; 3 Cologne Excellence Cluster on Cellular Stress Responses in Aging-Associated Diseases (CECAD), University of Cologne, 50931, Cologne, Germany; 4 Center for Molecular Medicine Cologne (CMMC), University of Cologne, 50931, Cologne, Germany; 5 Department of Pediatrics, University Medical Faculty Giessen, 35392, Giessen, Germany; 6 Institute of Neuropathology University Medical Faculty Giessen and Marburg, 35392, Giessen, Germany; Charité Universitätsmedizin Berlin, NeuroCure Clinical Research Center, GERMANY

## Abstract

Massively parallel sequencing of whole genomes and exomes has facilitated a direct assessment of causative genetic variation, now enabling the identification of genetic factors involved in rare diseases (RD) with Mendelian inheritance patterns on an almost routine basis. Here, we describe the illustrative case of a single consanguineous family where this strategy suffered from the difficulty to distinguish between two etiologically distinct disorders, namely the co-occurrence of hereditary hypophosphatemic rickets (HRR) and congenital myopathies (CM), by their phenotypic manifestation alone. We used parametric linkage analysis, homozygosity mapping and whole exome-sequencing to identify mutations underlying HRR and CM. We also present an approximate approach for assessing the probability of co-occurrence of two unlinked recessive RD in a single family as a function of the degree of consanguinity and the frequency of the disease-causing alleles. Linkage analysis and homozygosity mapping yielded elusive results when assuming a single RD, but whole-exome sequencing helped to identify two mutations in two genes, namely *SLC34A3* and *SEPN1*, that segregated independently in this family and that have previously been linked to two etiologically different diseases. We assess the increase in chance co-occurrence of rare diseases due to consanguinity, i.e. under circumstances that generally favor linkage mapping of recessive disease, and show that this probability can increase by several orders of magnitudes. We conclude that such potential co-occurrence represents an underestimated risk when analyzing rare or undefined diseases in consanguineous families and should be given more consideration in the clinical and genetic evaluation.

## Introduction

Rare diseases (RD) are defined as disorders that affect only a minor proportion of the population. The European Union considers a disease to be rare if fewer than 5 people per 10,000 are affected (http://ec.europa.eu/health/rare_diseases/), corresponding to a prevalence < 0.05%. Similarly, any disease with less than 200,000 cases is considered rare in the United States (US) (http://rarediseases.info.nih.gov/), corresponding to a prevalence of < 0.063% in a population of approximately 316 million (http://www.census.gov/). Although being rare on their own, the large number of RD (5–8000; http://ec.europa.eu/health/rare_diseases/) leads to substantial proportions of the population being affected with any one of them. It is estimated that about 6–8% of the total population of approximately 500 million (http://epp.eurostat.ec.europa.eu/) suffer from such disorders throughout the 27 member states of the European Union, and a similar percentage (7.9–9.5%, http://rarediseases.info.nih.gov/) has been reported for the US. Thus, RD pose a serious challenge to population health and warrant investigation of their etiology. Despite their low prevalence, there have been a number of reports on the co-occurrence of two independent recessive diseases in consanguineous and non- consanguineous families in the past (e.g. [1–7]).

Diseases that follow a recessive Mendelian inheritance pattern have been an early target for indirect statistical mapping approaches, using parametric and non-parametric linkage mapping as well as homozygosity mapping (e.g. [8–10] for cystic fibrosis). However, factors such as locus and allelic heterogeneity as well as unspecific symptomatology can blur a clear relationship between causative factor and disease and thereby decrease the statistical power to detect such factors. Failures to repeat early mapping successes with other disorders and ensuing frustration led to a re-orientation of statistical mapping approaches towards association studies [11]. Next-generation sequencing (NGS) technologies now facilitate a direct assessment of causative variation, thereby obliterating the need for indirect mapping approaches. This has led to a recent renaissance of linkage-based approaches. Linkage analysis of variants obtained from whole-exome sequencing (WES) is currently the method of choice for deciphering recessive RD. In particular, large families living in or stemming from rural regions where marriages between relatives are common provide favorable conditions for statistical mapping approaches, because increased consanguinity levels lead to increased prevalence of some recessive diseases and to higher statistical power in these populations [12].

Still, some issues remain. The elucidation of genetic causes involved in RD can also be hampered by factors such as variability in the phenotypic manifestation with respect to age of onset, clinical features, and severity of symptoms and from difficulties to distinguish two etiologically distinct disorders with partially overlapping symptoms [13]. Successful identification of disease-associated genetic loci critically depends on precise phenotype information as much as on correctly specified inheritance models. Indeed, problems in delineating the exact phenotype are generally correlated with failures in discriminating between affected and unaffected family members. Furthermore, a situation where two phenotypically similar recessive diseases segregate independently in a single family is usually not taken into consideration given the low prevalence of any of these two diseases, despite previous reports of a co-occurrence of rare Mendelian diseases.

An example of a rare Mendelian disease is congenital myopathy (CM), with a prevalence of 1/26000 in the US (www.orpha.net; Orpha number: ORPHA97242, [14]). CM includes a group of neuromuscular disorders with variable clinical and histopathological features, in which muscular weakness and skeletal deformities are prominent findings. Recent technical advance has revealed marked genetic heterogeneity, and has shown that specific genetic defects are associated with variable clinical and histopathological phenotypes [15].

Hereditary hypophosphatemic rickets (HHR) is a further RD with an incidence of 1/21 000 [16], encompassing several genetically distinct disorders, all characterized by hypophosphatemia secondary to renal phosphate wasting, rickets, short stature, and skeletal deformities. Muscular weakness is also frequently reported, and studies in mice and humans with HHR have shown altered muscle composition and function [17].

In this study, we present a straightforward approximate approach for estimating the probability for the co-occurrence of two unlinked recessive RD in a single family as a function of the degree of consanguinity in the population and the frequency of the disease-causing alleles. We show that consanguinity can increase this probability by several orders of magnitudes, thereby increasing the chances of a manifestation of two etiologically different recessive Mendelian diseases in a single family. We illustrate our approach with a consanguineous family where several members had muscular weakness of variable degree and abnormal muscle biopsy findings; along with skeletal deformities, early ventilatory failure, and renal phosphate wasting occurring in some of them. Linkage analysis of this family yielded elusive results when assuming a single segregating RD, but WES helped to identify two mutations in two genes, namely *SLC34A3* and *SEPN1*, that segregated independently in this family and that have previously been linked to two etiologically different diseases.

## Materials and Methods

### Statistical basis for calculation of the joint occurrence of two recessive diseases in consanguineous populations

Assume that the occurrence of a recessive disease can be, at least in parts, parameterized by the homozygous carrier-ship of a deleterious allele *A* at a particular locus. Let *q*_*A*_ denote the population frequency of this allele and let the genotype *AA* be fully penetrant, i.e. carriers of genotype *AA* will always become affected. With little or no selection before the reproductive phase, the probability of becoming affected for an individual randomly drawn from an outbred population, *P*(*D*), will equal the Hardy-Weinberg proportion of the genotype in the population:

$$P\left(D\right)=q_{A}^{2}$$ (1)

However, if consanguineous marriages are common in a population or subpopulation, the occurrence of the disease due to homozygous *AA* carrier-ship will increase. Its probability can then be parameterized by the inbreeding coefficient *F*_*I*_ [18], namely

$$P_{I}\left(D\right)=F_{I}\cdot q_{A}+\left(1-F_{I}\right)\cdot q_{A}^{2},$$ (2)

which reduces to Eq 1 for the case of no inbreeding (*F*_*I*_ = 0).

A lower limit for the probability that two or more diseases occur within the same family is the co-occurrence of these diseases in a single individual. More specific, if some recessive disease, *D*_1_, is caused by homozygous carrier-ship of allele *A* at one locus and another recessive disease, *D*_2_, by homozygous carrier-ship of allele *B* at another unlinked locus, then the probability of their joint occurrence is simply the product *P*_*I*_(*D*_1_)*P*_*I*_(*D*_2_). The increase in probability of a joint occurrence of both diseases in a single individual due to inbreeding can then expressed as a simple function of the allele frequencies, *q*_*A*_ and *q*_*B*_, and the inbreeding coefficient

$$\begin{array}{lll} f\left(F_{I},q_{A},q_{B}\right) & = & \frac{P_{I}\left(D_{1}\right)\cdot P_{I}\left(D_{2}\right)}{P\left(D_{1}\right)\cdot P\left(D_{2}\right)}\\ & = & \frac{F_{I}\cdot q_{A}+\left(1-F_{I}\right)\cdot q_{A}^{2}}{q_{A}^{2}}\cdot \frac{F_{I}\cdot q_{B}+\left(1-F_{I}\right)\cdot q_{B}^{2}}{q_{B}^{2}}\\ & = & \frac{F_{I}+\left(1-F_{I}\right)\cdot q_{A}}{q_{A}}\cdot \frac{F_{I}+\left(1-F_{I}\right)\cdot q_{B}}{q_{B}} \end{array},$$ (3)

which reduces to

$$f\left(F_{I},q\right)=\frac{{\left(F_{I}+\left(1-F_{I}\right)\cdot q\right)}^{2}}{q^{2}},$$ (4)

if one assumes, for the sake of simplicity, equal population frequencies of both alleles (*q* = *q*_*A*_ = *q*_*B*_). Note that the consideration of less than fully penetrant disease loci would not change expressions (3) and (4).

Furthermore, we may also inquire about the probability that at least two out of a set of *k* diseases co-occur in an individual. Again assuming unlinked and fully penetrant loci and allele frequencies *q*_1_, …, *q*_*k*_ for the different loci, respectively, the probability for this event can be approximated by

$$P\left(c\geq 2|F_{I},q_{1},\ldots ,q_{k}\right)=1-\overset{k}{\underset{i=1}{\prod }}\left(1-q_{i}^{I}\right)-\overset{k}{\underset{i=1}{\sum }}\left(q_{i}^{I}\overset{k}{\underset{j=1,j\neq i}{\prod }}\left(1-q_{j}^{I}\right)\right),$$ (5)

where $q_{i}^{I}$ denotes the frequency of the *i*-th homozygous genotype under consanguinity:

$$q_{i}^{I}=F_{I}q_{i}+\left(1-F_{I}\right)\cdot q_{i}^{2}.$$ (6)

### Phenotypic characterization of family members suspected to suffer from a not yet described congenital myopathy with hypophospathemic rickets

**Fig 1:**
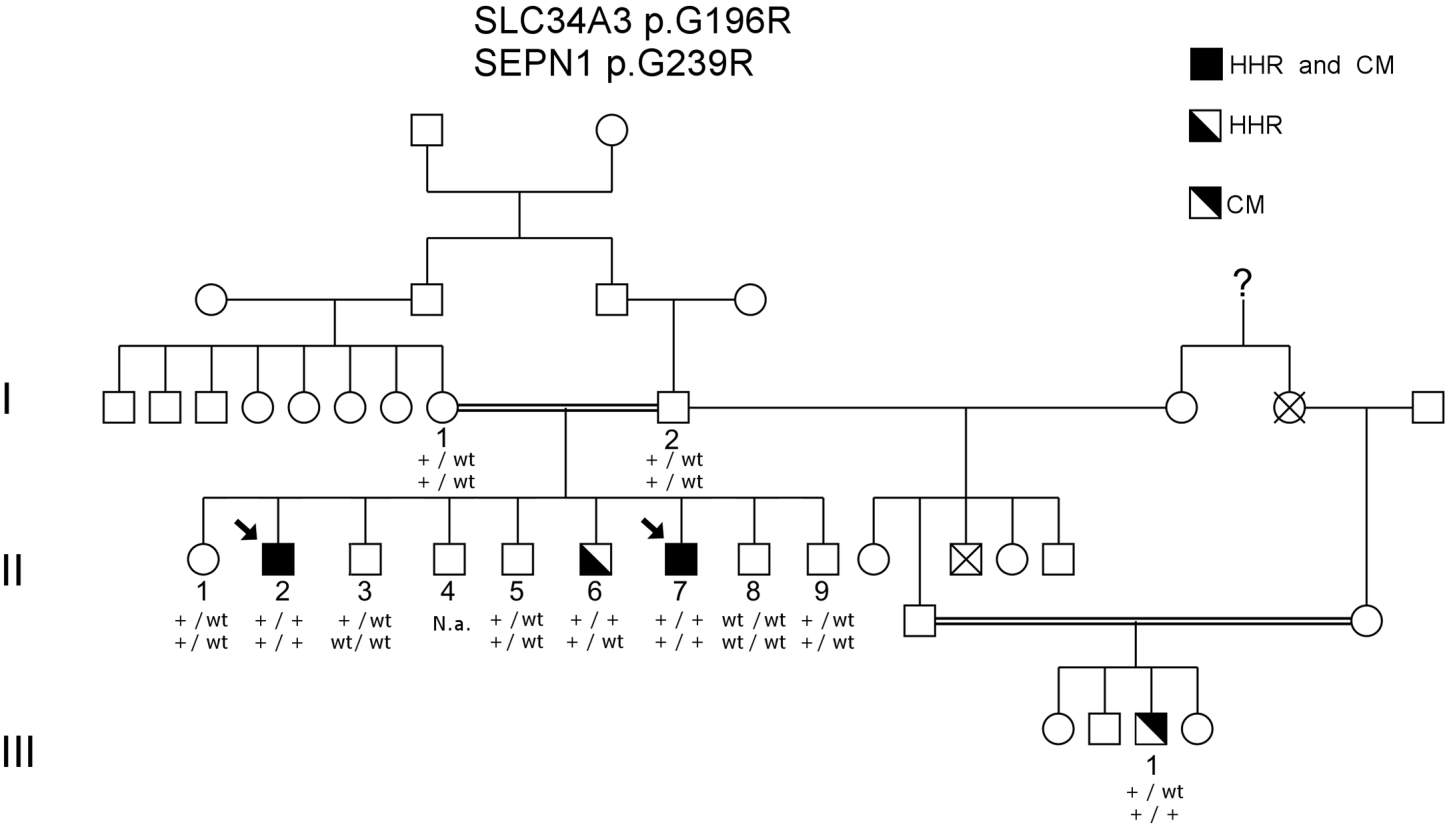
Pedigree and mutation segregation. All patients exhibiting symptoms of HHR are homozygous for the mutation *SLC34A3* p.G196R (c.586G>A NM_080877.2), whereas all patients expressing symptoms of CM are homozygous for the mutation *SEPN1* p.G239R (c.715G>A NM_206926.1). Therefore, patients II-2 and II-7 present features of both diseases. Arrows indicate index patients. Abbreviations: HHR = Hereditary hypophosphatemic rickets; CM = congenital myopathy.

**Fig 2:**
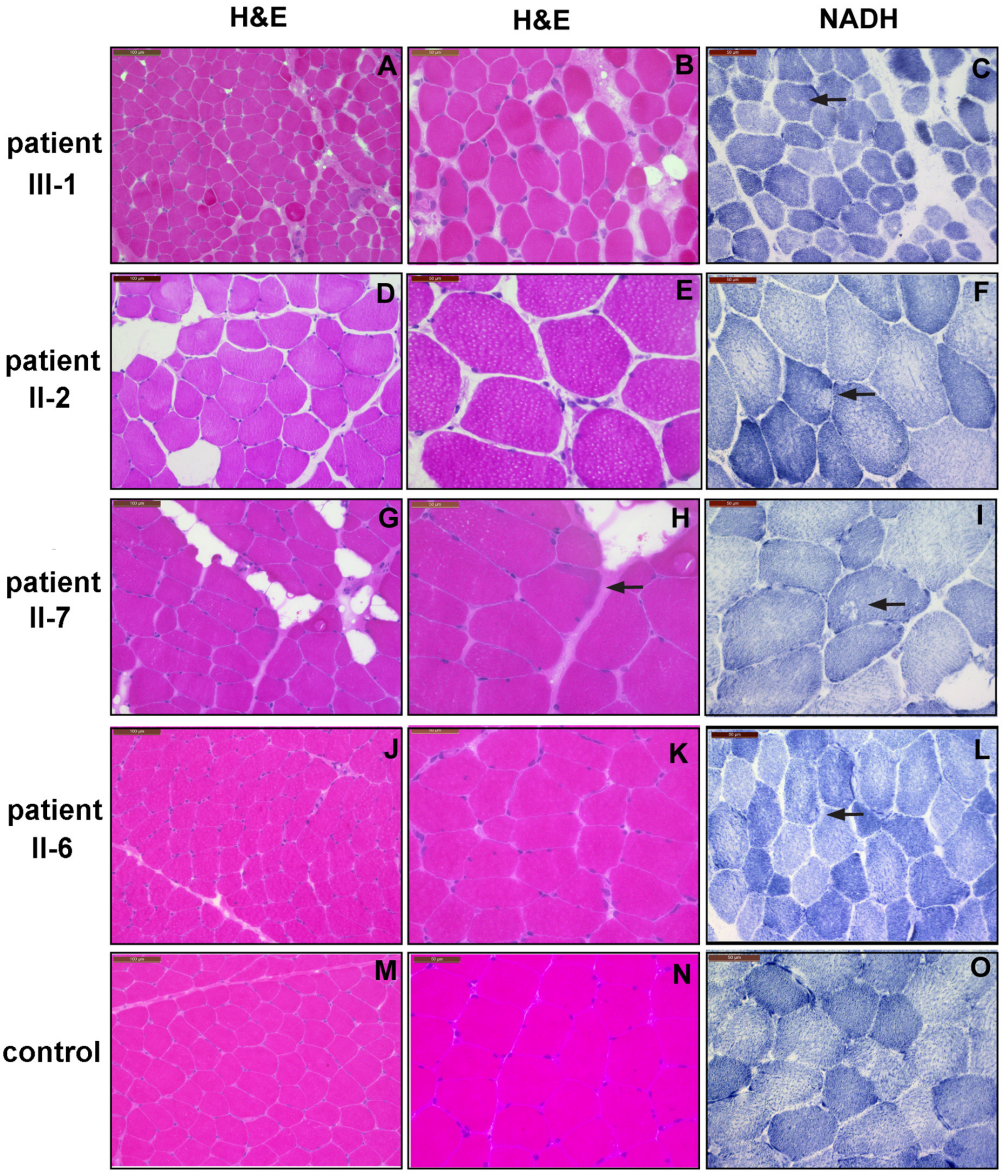
Histochemical and immunohistochemial biopsy analyses from affected family members and a normal control. H&E staining of transverse cryostat sections displays findings well compatible with a slowly progressive myopathy with some replacement of muscle by fat in patients III-1 (A), II-2 (D), and II-7 (G); and is also abnormal in patient II-6 due to increased fiber variability (J) compared to a control (M). There is increased variability in fiber size in all family members (B,E,H,K) Notice also additional small atrophic fibers in patient II-2 (E), and some subsarcolemnal muscle fiber disorganization in patient II-7 (H arrow). NADH enzymehistochemistry depicts core like lesions in patients II-2 (F) and II-6 (I), and minor myofibrillar disorganization in patients III-1 (C) and II-6 (L).

We identified a consanguineous Turkish family with several members affected to a variable degree with muscular weakness and hypotonia in conjunction with early ventilatory failure and/or hypophosphatemic rickets in some of them (Fig 1). The institutional review board (IRB) of the Department of Medicine, University of Giessen, Germany, approved this study. Written informed consent for genetic analysis was obtained from all subjects or their legal guardians according to study protocols approved by this IRB. Clinical and paraclinical findings of the affected individuals are summarized in Table 1, and muscle biopsy findings are shown in Table 2, Fig 2, and S1 Fig.

**Table 1:** Synopsis of clinical and paraclinical findings in patients II-2, II-6, II-7 and III-1.

	II-2	II-6	II-7	III-1
*SEPN1* mutation	**+**	**-**	**+**	**+**
*SLC34A3* mutation	**+**	**+**	**+**	**-**
Delayed motor milestones	+	-	-	+
Muscular weakness	+	+	+	+
Muscular hypotonia	+	+	+	+
Rigid spine	+	-	-	-
Short stature	+	-	-	-
X-legs	+	+	+	-
Ventilatory failure (years)	22	-	-	8
Creatine kinase (12–174 U/l)	51	80	78	116
Abnormal EMG	+	-	+	-
Abnormal muscle biopsy	+	+	+	+
Loss of ambulance	-	-	-	-
Rickets	+	+	+	-
Serum calcium (2.0–2.6 mmol/l)	2.4	2.4	2.6	2.1
Serum phosphate(1.2–1.8 mmol/l)	**0.7**	**0.9**	**1.0**	1.4
Alkaline phosphatase (92–390 U/l)	**960**	**562**	271	251
Urine calcium/creatinine ratio (<0.3)	**0.76**	**1.13**	**0.59**	0.18
Phosphate re-absorption (82–90%)	**15**	**26**	**43**	82
1,25- dihydroxy-cholecalciferol (18–62 pg/ml)	21	60	111	45

Numbers in brackets correspond to normal and bold values to abnormal values

**Table 2:** Muscle morphology in patients II-2, II-6, II-7 and III-1.

	II-2	II-6	II-7	III-1
Age of biopsy (years)	24	17	15	4
Biopsied muscle	vastus lat.	vastus lat.	vastus lat.	vastus lat.
Genetic phenotype	CM+HHR	HHR	CM+HHR	CM
Variability of fiber size	++	(+)	++	+
Internal nuclei	(+)	-	(+)	(+)
Core like lesions (NADH)	++	(+)	+	(+)
Endomysial Fibrosis	(+)	-	(+)	(+)
Fatty replacement	++	-	++	+
Inclusions (Desmin)	+	-	++	(+)
Inclusions (αβ Crystallin)	-	-	(+)	-
Typ II fiber predominance	+	+	+	(+)
Fiber necrosis	-	-	-	-
Fiber regeneration	-	-	-	-
CD56 positive fibers	-	-	+	+
Endomysial lymphozytes	+	-	-	-
Minicores (EM)	+	-	+	-

EM = electron microscopy

**Patient III-1** was referred at age 3 ½ years because of delayed motor development and muscular hypotonia. His height was between the third and 10^th^ percentile. He was able to stand up from the floor and to climb stairs, but had marked difficulties to sit up from the supine position. Mobility of the spine was normal, and his creatine kinase (CK) level was within the normal range. Electromyography (EMG) showed no unequivocal abnormalities, but a muscle biopsy demonstrated myopathic changes. Determination of calcium, phosphate, 1,25-OH-dihydrocholecalciferol, and tubular phosphate re-absorption revealed normal values. A diagnosis of CM was made and regular follow-up was recommended, but appointments were not kept by the family. At age 8 years, the boy was admitted to our hospital as an emergency case with pneumonia and acute ventilatory failure, necessitating implementation of permanent non-invasive nocturnal ventilation.

In **patient II-2**, mild muscular weakness and reduced endurance were noticed since early childhood. The diagnosis of HHR was made at age 8 years due to dysproportionately short statue, and skeletal deformities such as rickets, flat occiput, and x-legs; in conjunction with hypophosphatemia and reduced tubular phosphate re-absorption. Mutations in *PHEX1*, and during the following years also in *FGF23*, *DMP1*, *and CLCN5* were ruled out. Muscular weakness initially improved during treatment with calcium and rocaltrol, and therefore was attributed to HHR. But after the age of 20 years, he became increasingly short of breath, and non-invasive ventilation had to be started at age 22 years. A muscle biopsy taken at this time showed mild to moderate myopathic changes. At age 26 years, his height was 5 cm below the third percentile. He was able to rise up from the floor, walked with a waddling gait, and was still climbing stairs. He had a rigid spine and bending of the head is nearly completely abolished.

**Patient II-6**’s early motor development was delayed from the beginning. The diagnosis of HHR was established at age 6½ years, after the disease had been diagnosed in his older brother. Muscular hypotonia and weakness were documented from this age on. At age 13 years, his CK and EMG were normal, while a muscle biopsy taken during an osteotomy to correct his x-legs revealed single muscle fiber atrophy. At last follow-up, at age 15 years, he showed a mild proximal muscle weakness, and his height was on the 25^th^ percentile.

In **patient II-7**, HHR was diagnosed at age 3 ½ years together with his older brothers. He also suffered from mild proximal muscle weakness. At age 13 years, his CK was normal, while the EMG showed a myopathic pattern. A muscle biopsy performed during a metal removal secondary to a femoral fracture showed myopathic changes. At last follow-up, at age 17 years, his height was on the 50^th^ percentile. He walked with a waddling gait and was unable to run fast, but stood up from the floor without Gower’s sign, and climbed stairs. Bending of his spine was not limited. He had difficulties to sit up, while his vital capacity is within the normal range.

### Genotyping

DNA samples from the core family members I-1, I-2, II-1–II-3, and II-5–II-9 (Fig 1) were genotyped for linkage analysis using Illumina HumanLinkage-12 BeadChip, consisting of 6,090 single nucleotide polymorphism (SNP) probes according to the manufacturer's instructions. The genotype assignments were determined with the GenomeStudio version 1.9.4 genotyping module (Illumina Inc., San Diego, CA). All samples passed quality control (Array SNP call rate > 99%). Downstream quality control was automatically performed by the Superlink tool (for details see [19]) including marker filtering, linkage disequilibrium (LD) pruning as well as Mendelian error checking.

### Initial mutational screening strategy

Given the family structure (Fig 1) and phenotype manifestations, we initially assumed an autosomal recessive mode of inheritance with incomplete penetrance for a novel rare disease in the family.

### Initial mapping strategies

We pursued two mapping strategies based on the obtained SNP data. First, we performed genome-wide parametric linkage analysis under a recessive model of inheritance. To this end, we assumed a single locus, first with complete penetrance, and determined all non-healthy family members as affected, thereby assuming all affected members to suffer from the same rare disease. Two- and multi-point LOD scores were calculated with 99% penetrance using Superlink [19]. Second, we performed genome-wide homozygosity mapping, using HomozygosityMapper2012 [20].

### Sequence capture and next generation sequencing

Since we expected at the beginning the segregation of a single disease, we generated exome sequencing data for patients II-2 and II-7 (Fig 1), both expressing the full range of symptoms observed in this family. We fragmented 1 μg of DNA from each of the two patients and their healthy parents with sonification technology (Covaris, Woburn, MA, USA). The fragments were end-repaired and adaptor-ligated, including incorporation of sample index barcodes. After size selection, we subjected the library to an enrichment process with the SeqCap EZ Human Exome Library version 2.0 kit (Roche NimbleGen, Madison, WI, USA). The enriched pools were then sequenced on one lane of an Illumina HiSeq 2000 sequencing instrument (Illumina, San Diego, CA, USA) with a paired-end 2×100 bp protocol. We filtered primary data according to signal purity with the Illumina Realtime Analysis software version 1.8. Subsequently, we mapped the reads to the human genome reference build hg19 with the ELANDv2 alignment algorithm on a multinode compute cluster. By use of CASAVA version 1.8 (Illumina processing software, Illumina), PCR duplicates were filtered out and the output was converted into BAM format.

An average coverage of >30x was achieved for 77% of the target sequence. Given the consanguinity of the family, variants were filtered for high-quality rare homozygous variants by comparison with external reference sources, including an in-house variation database (N> 1300 exomes) and more than 60,000 individuals without Mendelian disorders from the Exome Aggregation Consortium (ExAC; www.exac.broadinstitute.org; accessed Oct. 2015). We excluded those variants that had an allele frequency exceeding 1% in any of these sources from further investigation. The segregation was tracked *in silico* by haplotypes identified in the mapping analysis and then validated by Sanger sequencing. The Sanger sequencing was performed following standard protocols.

## Results

We identified a consanguineous Turkish family with several members being affected to a variable degree with muscular weakness and hypotonia in conjunction with early ventilatory failure and/or hypophosphatemic rickets in some of them (Fig 1). Clinical and paraclinical findings of the affected individuals are summarized in Table 1, and muscle biopsy findings are shown in Table 2, Fig 2, and S1 Fig (see also Materials and Methods).

### Whole-genome linkage analysis

**Fig 3:**
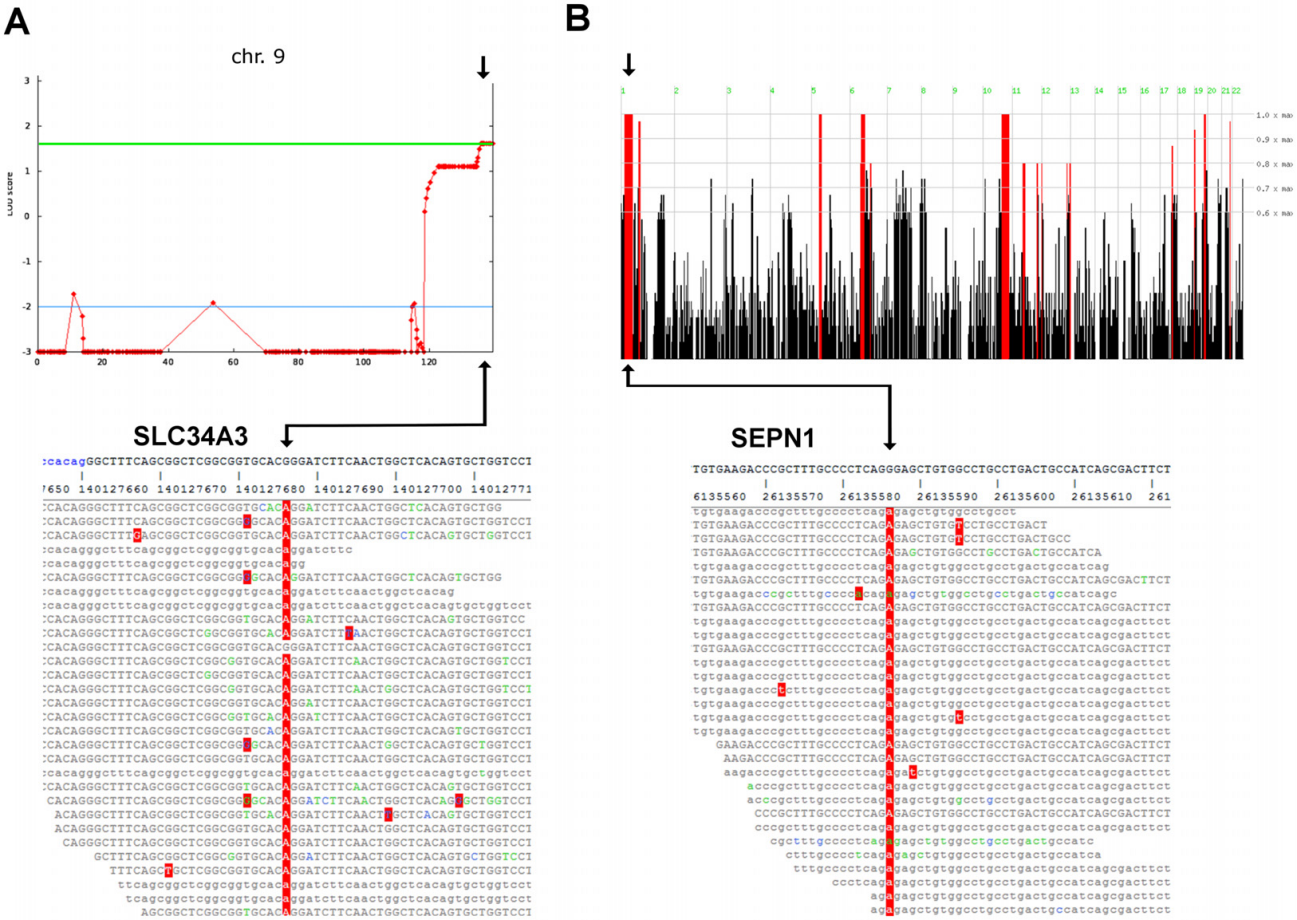
Variant discovery. **(A)** Top: The *SEPN1* p.G239R (c.715G>A NM_206926.1) mutation is located in a large, continuous segment of homozygosity identified in patients II-2 and II-7 of 33Mb (chr1:22Mb-55Mb). Bottom: Schematic representation of the mapped sequencing reads of the p.G239R mutation. **(B)** Top: Linkage analysis of all patients with symptoms of HHR (II-2, II-6 and II-7) yielded maximum genome-wide two-point and multi-point LOD score of 1.62 on 9q34, indicated by green line, harboring the *SLC34A3* p.G196R (c.586G>A NM_080877.2) mutation identified in II-2 and II-7. Bottom: Schematic representation of the mapped sequencing reads of the *SLC34A3* c.586G>A (NM_080877.2) variant.

An initial parametric linkage analysis was performed under the assumption of a single recessive disease with 99% penetrance and failed to identify a shared homozygous haplotype in all patients. Thereafter, linkage analysis was performed including all family members with symptoms of HHR (II-2, II-6 and II-7). This yielded a maximum genome-wide two-point and multi-point LOD score of 1.82 on 9q34 (Fig 3A). At this point, the muscle biopsies were re-assessed. Re-evaluation confirmed unequivocal myopathic features in patients II-2, II-7, and III-1, whereas the muscle histology from patient II-6 was interpreted as showing abnormal, but unspecific changes. In line with this observation we identified a homozygous p.G196R mutation (c.586G>A, NM_080877.2; p.G196R) in the *SLC34A3* gene in the three siblings affected by HHR.

### Sequencing of a shared homozygous region

In addition, we performed homozygosity mapping in patients II-2 and II-7, who displayed signs of HHR and unequivocal myopathic features in the muscle biopsy, hypothesizing an independent molecular mechanism of this specific phenotype. The analysis revealed five large, continuous segments of homozygosity present in both patients and heterozygous in the parents. The second largest segment of 33Mb (chr1: 22Mb-55Mb; Fig 3B) contained the *SEPN1* gene, known to be associated with different forms of CM [21]. Consecutively, WES revealed the presence of a homozygous p.G239R mutation in *SEPN1* (c.715G>A, NM_206926.1, p.G239R) in both affected siblings. The p.G239R exchange had been previously reported by Moghadaszadeh et al. [22] in a patient with rigid spine muscular dystrophy. However, the variant had been falsely reported in their paper and has been corrected and annotated in HGMD (www.hgmd.cf.ac.uk) recently. The correct annotated variant exchange is (GRCh37: Chr1:26135586, NM_206926,NP_996809.1: p.Gly239Arg) and therefore identical with ours.

Although a total of 42 homozygous variants were identified in the whole coding region shared by both sequenced individuals with HHR and CM, no other homozygous variants were detected in genes that are known to relate to CM or HHR (S1 Table). Indeed, both detected mutations, p.G196R in *SLC19A3* and p.G239R in *SEPN1*, had been previously reported in patients with either HHRH [23] or rigid spine muscular dystrophy-1 (RSD-1) [22], respectively.

Finally, Sanger sequencing confirmed heterozygous carrier-ship of the *SLC19A3* and *SEPN1* mutations in the clinically healthy parents (I1 and I2), while patients II-3 and II-7 were found to be homozygous for both pathogenic variants. Furthermore, patient II-6 was found to be homozygous for the *SLC34A3* only, while patient III-1 carried merely the homozygous *SEPN1* mutation (Fig 3).

### Co-occurrence of two rare recessive diseases

Given the refined phenotype assessment in our sample family, we theoretically explored the probability of a joint occurrence of two unlinked, etiologically different diseases in a single family and assessed how consanguinity impacts on this probability. To this end, we simplified the task to the question of how likely it is that two particular independent diseases co-occur in a single individual, thereby approximating the probability for more complex cases, such as the co-occurrence in two related family members (see Materials and Methods).

**Fig 4:**
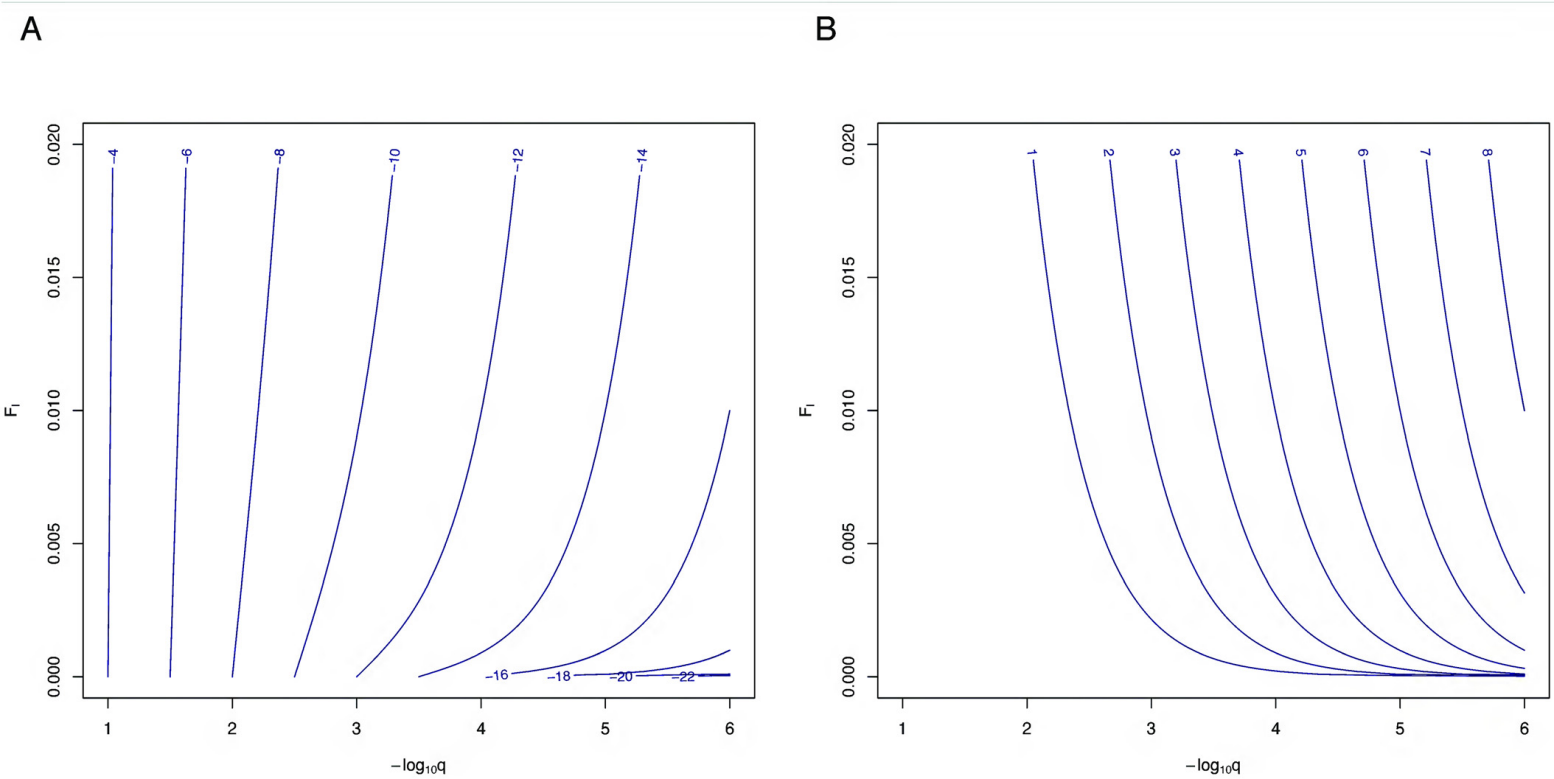
Co-occurrence of two recessive diseases under consanguinity. Probabilities are calculated for a model of two recessive diseases, each being caused by a single fully penetrant locus with identical susceptibility allele frequency, q, and both loci being unlinked. **(A)** Log_10_ value of the probability to observe two recessive diseases in a single family by chance as a function of consanguinity level *F*_*I*_ in the general population and the same risk allele frequency, q, at both unlinked loci. **(B)** Log_10_ value of the increase in the co-occurrence probability due to consanguinity compared to the non-consanguineous case (*F*_*I*_ = 0). Probabilities and allele frequencies are depicted using their decadic logarithm (log_10_). For example, when assuming values of *F*_*I*_ = 0.01 and q = 0.001 = 10^−3^, the two diseases will jointly occur in a single family with a probability of roughly 10^−10^, which equals approximately an 10^2^ = 100-fold increase in probability in comparison to a non-consanguineous population.

Assuming the same frequency, *q*, for the causative alleles at both disease-related loci and applying Eq 4, the probability of the joint occurrence of both diseases (Fig 4A) and the increase in probability due to consanguinity (Fig 4B) can be plotted as a function of *q* and the inbreeding coefficient *F*_*I*_. For relatively common alleles of frequencies > 1%, the co-occurrence probability is largely determined by *q* and, correspondingly, increased consanguinity levels barely lead to an increase of this probability. With increasingly rare alleles, the impact of consanguinity grows steeply, making the co-occurrence of two diseases, although very rare in absolute terms, more likely by several orders of magnitude. For example, values of 0.01 for *F*_*I*_ would lead to an ~120-fold probability increase for *q* = 0.001 and even ~10,200-fold increase for *q* = 0.0001. With more pronounced consanguinity (*F*_*I*_ = 0.05), the corresponding numbers would equal ~2,600 and ~251,000, respectively.

Considering the particular case of HHR und CM, we may also roughly assess the increase in probability to observe both diseases together in a single individual, assuming unlinked causal loci for these two diseases. To this end, we postulated that each of the diseases is exclusively caused by a single, fully penetrant mutation under a recessive disease model and assumed an average inbreeding coefficient of *F*_*I*_ = 0.01 for eastern Turkey (http://consang.net/), causing an only modest deviation of the genotype frequencies from Hardy-Weinberg equilibrium in absolute terms. Incidence values of 3.9/100,000 and 5.0/100,000 for HHR [16] and CM [14], respectively, then lead to frequency alleles estimates of 0.0030 and 0.0037, respectively, using the solution to the quadratic Eq 2. Applying Eq 3, the probability of observing both diseases together is then increased by factor ~16, compared to the case of a non-inbred population. We caution that this calculation represents only a rough approximation of the probability of disease co-occurrence, given that a number of other factors may also play a role in the etiology of both diseases.

We also considered the case where a set of *k* unlinked diseases show overlap of clinical symptoms that are difficult to distinguish and inquired how likely the co-occurrence of any two of them is (see Materials and Methods). Again, we assumed the same frequency, *q*, for the causative alleles at all disease-related loci, applied Eq 6 and considered in turn a set of 3, 5 and 10 diseases. Neither the probability of co-occurrence of at least two of these diseases, nor the increase in probability due to inbreeding did substantially differ from the case of two particular diseases (data not shown). This observation is likely due to the extremely small probability for more than two diseases to co-occur, being magnitudes smaller than the probability of co-occurrence of just two diseases and therefore contributing only little probability mass to the calculations.

## Discussion

Recent advances in sequencing technology have raised high hopes for identifying genetic causes of many RD that had not been amenable to classic indirect linkage mapping or population-based association studies in the past. Indeed, many causative *de-novo* mutations and rare variants implicated in familial disease forms have been discovered in the past few years, sometimes based on family data that were sampled many years ago (e.g. [24]). However, a seemingly straightforward analysis can be hampered by several blurring factors. This includes incomplete penetrance of causative factors, locus and allelic heterogeneity as well as phenotypic heterogeneity, but also overlap of clinical symptomatology, and unspecific paraclinical features. Consanguineous pedigrees are a favorable sampling design for studying rare recessive diseases due to increased prevalence of some diseases and a higher statistical power for identifying causative genetic factors, and, thus, populations with increased levels of consanguinity have primarily been targeted for studying such diseases. However, these populations are precisely those where one would expect to see a much higher co-occurrence of rare recessive diseases by chance than in a completely non- consanguineous population. If these diseases show overlapping symptomatology, it is quite likely that they will be analyzed as a single disease in the first place and that the induced “locus heterogeneity” will hamper any successful linkage analysis. As we have shown here, the probability of co-occurrence can increase by several orders of magnitude, depending on the frequency of the risk allele and the degree of consanguinity. We note that the increase in co-occurrence probability is particularly severe for such rare and very rare variants. We therefore suggest that studies on rare recessive diseases should put strong emphasis on precise phenotyping and should consider the possibility of co-occurrence of two rare and phenotypically similar diseases within a single family especially when sampling from consanguineous populations.

As an instructive illustration, we presented the genetic analysis results of a consanguineous family with several members erroneously assumed to suffer from a yet not described recessive form of CM with renal phosphate wasting as an additional feature. Instead, linkage analysis combined with WES revealed independent segregation of two rare genetically distinct disorders, *SEPN1*-related myopathy and *HHRH*, both causing muscular weakness and abnormal muscle histology.

In this family, three affected members harbored a homozygous, previously reported mutation in *SEPN1*, coding selenoprotein N, a glycoprotein-localized within the endoplasmic reticulum [25]. As in our family, defects in *SEPN1* have been associated with a variable clinico-histopathological picture that includes rigid spine muscular dystrophy (RSMD-1), multiminicore disease, desmin-related myopathy with Mallory body-like inclusions (MR-DRM) [26, 27], and also non-specific myopathological features [28]. Retrospectively, some findings like rigid spine in patient II-2, early ventilatory failure despite preserved ambulance in subjects II-2 and III-1, core-like lesions in patients II-2 and II-7, and fibers showing minicores or expressing αβ-Crystallin in subject II-7 are consistent with a myopathy caused by *SEPN1* defects [26, 28–30]. However, the clinical symptoms and histopathological features observed in affected family members may also well be found in other neuromuscular disorders, and, thus, seemed us not sufficient to identify *SEPN1* as a candidate gene of high priority.

Mutations in *SLC34A3*, coding the renal sodium-phosphate co-transporter NaPi-IIc have been recently identified as a further rare cause of HHR [31]. As in other forms of HHR muscular weakness has been noted [31, 32]. The exact reasons why muscle function is impaired in patients with HHR are unclear. But assessment of lower extremity muscle function and determination of calf muscle cross-sectional area and density by peripheral quantitative computed tomography has revealed lower muscle force and reduced muscle density in patients with and without limb deformities compared to controls [32]. Of note, the subject with the earliest onset of ventilatory failure was not affected by HHR, indicating that co-segregation of both genetic defects did not result in a more severe phenotype.

## Conclusions

Taken together, the findings in this family demonstrate the difficulties in defining the affection status in patients with CM, show the problems in differentiating specific types of CM, and illustrate that an inheritance model assuming the occurrence of two genetically distinct disorders should not be neglected in larger consanguineous families.

Targeted massive parallel sequencing and candidate gene screening combined with haplotype tracking was performed to identify the underlying genetic cause for the disease spectra in this family. Our analysis unmasked that the phenotype in the family did not reflect a complex novel disease, but instead was due to two homozygous mutations in *SEPN1* and *SLC34A3*, respectively. [22, 23].

In retrospect, the event of exhibiting a combined recessive *SLC34A3* and *SEPN1* disease is more likely than expected initially, since in consanguineous unions autosomal recessive disorders are increased [33, 34], and because the p.G196R (*SLC34A3*) and the p.G239R (*SEPN1*) alleles were identified in Turkish and Iranian families, suggesting a higher frequency of the variants in middle eastern populations. Thus, in the clinical diagnostic procedure the presence of two rare diseases in one family should be taken into account, in particular if the disease risk is increased. Therefore, analysis of all known disease symptom associated genes should be considered, especially in clinically heterogeneous disorders like CM, where diagnosis is challenging due to phenotypic variability [35–38].

In the family reported, the final diagnosis could only be established by genetic analysis. The use of WES for clinical diagnostics offers a rapid and inexpensive opportunity and will improve genetic counseling in Mendelian diseases, even if masked as in the family presented in this report.

## Supporting Information

S1 Fig Histochemical, immunohistochemical, and ultrastructural muscle biopsy analyses of affected family members and a normal control. The muscle biopsies from all family members show type II fiber predominance (A,F,K,P) compared to a control (U). Immunohistochemical analyses display few myofibers expressing CD56 in patients III-1 (B, arrow) and II-7 (L, arrow). Small Desmin positive inclusions are present in patients III-1 (C, arrow), II-2 (H, arrow), and II-7 (M, arrow). αβCrystallin inclusion was only occasionally detected in patient II-7 (N, arrow). Ultrastructural analysis demonstrates unspecific myofibrillar disorganization with empty vacuoles in patients III-1 (E), II-2 (J) and II-6 (T) and Z-band streaming in patient II-7 (O, arrow).
(TIF)

S1 Table Exome sequencing results. List of rare (MAF<0.1) recessive mutations present in both patients II-2 and II-7 (Fig 1) who presented the full range of symptoms observed in this family.
(XLSX)
