# Supplementary figures and images for: Increased Probability of Co-Occurrence of Two Rare Diseases in Consanguineous Families and Resolution of a Complex Phenotype by Next Generation Sequencing
Source: PLoS One. 2016 Jan 20;11(1):e0146040. doi: 10.1371/journal.pone.0146040 (PMC4720433; doi:10.1371/journal.pone.0146040)

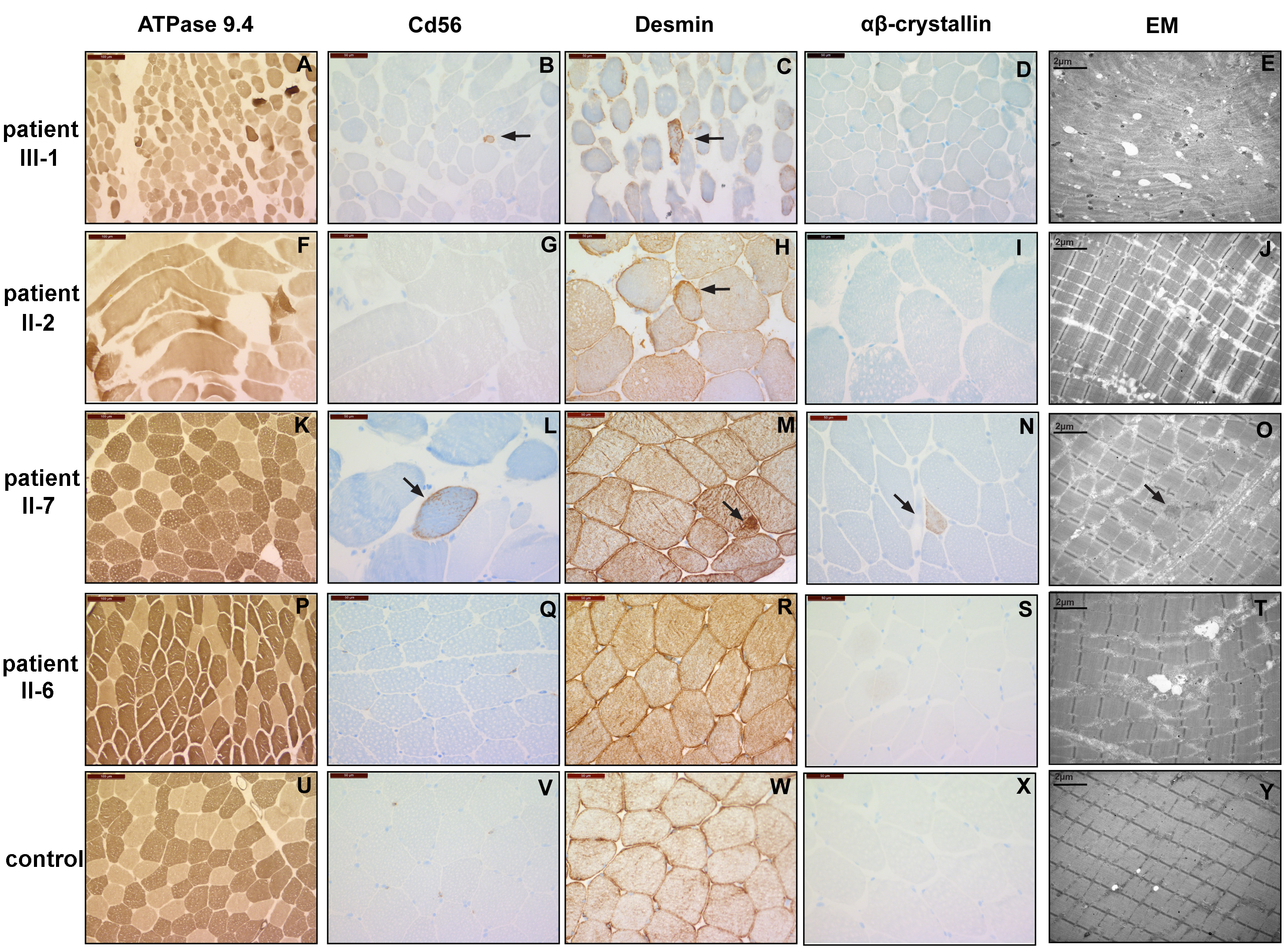

Supplement: S1 Fig — The muscle biopsies from all family members show type II fiber predominance (A,F,K,P) compared to a control (U). Immunohistochemical analyses display few myofibers expressing CD56 in patients III-1 (B, arrow) and II-7 (L, arrow). Small Desmin positive inclusions are present in patients III-1 (C, arrow), II-2 (H, arrow), and II-7 (M, arrow). αβCrystallin inclusion was only occasionally detected in patient II-7 (N, arrow). Ultrastructural analysis demonstrates unspecific myofibrillar disorganization with empty vacuoles in patients III-1 (E), II-2 (J) and II-6 (T) and Z-band streaming in patient II-7 (O, arrow). (TIF) [file pone.0146040.s001.tif]
